# Supplementary material for: A Shortcut from Metabolic-Associated Fatty Liver Disease (MAFLD) to Hepatocellular Carcinoma (HCC): c-MYC a Promising Target for Preventative Strategies and Individualized Therapy
Source: Cancers (Basel). 2021 Dec 31;14(1):192. doi: 10.3390/cancers14010192 (PMC8750626; doi:10.3390/cancers14010192)
Supplement: Supplementary file 1 [file cancers-14-00192-s001.zip › cancers-1482711-supplementary.pdf]

Supplementary materials: A Shortcut from Metabolic-Associated Fatty Liver Disease (MAFLD) to Hepatocellular Carcinoma (HCC): c-MYC a Promising Target for Preventative Strategies and Individualized Therapy

Feifei Guo, Olga Estévez-Vázquez, Raquel Benedé-Ubieto, Douglas Maya-Miles, Kang Zheng, Rocío Gallego-Durán, Ángela Rojas, Javier Ampuero, Manuel Romero-Gómez, Kaye Philip, Isioma U. Egbuniwe, Chaobo Chen, Jorge Simon, Teresa C. Delgado, María Luz Martínez-Chantar, Jie Sun, Johanna Reissing, Tony Bruns, Arantza Lamas-Paz, Manuel Gómez del Moral, Marius Maximilian Woitok, Javier Vaquero, José R. Regueiro, Christian Liedtke, Christian Trautwein, Rafael Bañares, Francisco Javier Cubero and Yulia A. Nevzorova

1. Supplementary Methods

1.1. Animal Diets

WD enriched in fat (40% kcal, Primex partially hydrogenated vegetable oil shortening), fructose (22% by wt), and cholesterol (2% by wt) (Research Diets, New Brunswick, NJ, cat. no. D09100301) [1] for 24 and 40 weeks; regular rodent chow diet (LASQC diet® Rod18-H, Altromin, Lage, Germany) was used as control diet. The comparation of chow and WD is shown in Table S3.

Chow diet enriched in 0.1% metformin was administrated for 20 weeks. Pure metformin was obtained from Sigma-Aldrich (St. Louis, MO, D150959) and mixed to homogeneity during manufacturing of the diets (C19051603, Research Diets, New Bunswick, NJ). The diets used in the research are shown in Table S4.

1.2. Glucose Tolerance Test (GTT)

At the experimental time-point (36 weeks old), GTT was performed after 6 h fasting through an intraperitoneal (*I.P.*) injection of 7.5 g/kg body mass of 20% glucose solution (Braun GmbH, Krönberg, Germany). Glucose levels were measured using an Accu-Check glucometer (Roche, Mannheim, Germany) at 0, 5, 30, 60, and 90 min after the injection as described [2].

1.3. Insulin Tolerance Test (ITT)

At the experimental time-point (36 weeks old), ITT was performed after 6 h of fasting through an intraperitoneal (I.P.) injection of 0.75 UI/kg body mass of insulin (Novo Nordisk, Barcelona, Spain) in 0.09% saline solution. Glucose levels were measured using an Accu-Check glucometer (Roche, Mannheim, Germany) at 0, 5, 30, 60, 90 min after the injection as describe [2].

1.4. Transmission Electron Microscopy

Fresh liver tissue was fixed in 4% PFA and 2.5% glutaraldehyde buffer at 4 °C. After washing the next day samples were post-fixed in 1% osmium tetroxide and dehydrated in different concentrations of acetone. Tissue was embedded in Spurr resin. Sample blocks were cut by an ultramicrotome and analysed using a Transmission Electron Microscope Jeol 1010 (Jeol, Peabody, USA).

1.5. Histological Analysis

Liver and eWAT histology were assessed by haematoxylin and eosin (H&E) and Sirius Red (SR) staining in 5 μm paraffin embedded sections using the standard protocols.

The presence of steatosis was further confirmed by Oil Red O staining in 7 μm frozen sections and quantified as previously described **[3]**.

1.6. Immunofluorescence (IF) Staining

Immunofluorescence F4/80, CD45 and Ki-67-staining was performed in 5 μm frozen liver sections as described [4] before DAPI (VectorLab, Burlingame, USA) was used to counterstain. F4/80 IF staining in eWAT was performed in paraffin sections as previously described [5]. The primary and secondary antibodies were shown in Table S5.

1.7. Immunohistochemistry (IHC)and Immunofluorescent (IF) Staining

Hepatocytes proliferation were identified through Ki-67 staining. Activated hepatic stellate cells (HSCs) were identified through α-smooth muscle actin (αSMA) staining in 5 μm liver sections as previous described [4]. Extensive c-MYC nuclear expression and Glutamine synthetase (GS) in the liver tissue evaluated by c-MYC and GS staining respectively in 5 μm liver sections (Table S6).

1.8. Image Analysis

Photomicrographs of stained sections were randomly taken at 40×, 100× and 200× total magnification with an optical or fluorescence microscope as appropriate. Eight photos from each mouse were quantified. Positive areas were determined using Image J software (http://imagej.nih.goc/ij/, accessed on 16.December 2021. National Institutes of Health, Bethesda, MD).

1.9. Triglycerides Quantification

50 mg liver pieces were homogenized using lysis buffer following the standard procedure. Triglycerides liquicolor mono kit was used to determine the concentration of triglycerides in the liver using a colorimetric assay (Human Diagnostics, Wiesbaden, Germany).

1.10. RNA Isolation and RT-qPCR

RNA was isolated from cryopreserved liver tissue using trizol (TermoFisher, Madrid, Spain) as described recently [6]. Pure RNA was transferred to cDNA through High Capacity cDNA Reverse Transcription Kit (ThermoFisher, Madrid, Spain) and mRNA relative expression to GAPDH was analysed by qPCR by using a Real Time PCR System 7300 (Applied Biosystems) and Fast SYBR Green Master Mix qPCR.

Relative expression was calculated using the $2^{-\Delta\Delta Ct}$ quantification formula normalizing each gene with the expression of Glycerinaldehyde-3-phosphate-Dehydrogenase (GAPDH), using as an internal standard**[7]**. The primer sequences are shown in **Table S7**.

1.11. Microarray Analysis

Concentrations and purity of RNA samples were determined on a NanoDrop spectrophotometer (TermoFisher, Madrid, Spain) according to the manufacturer's protocol. 2100 Bioanalyzer Instrument-Agilent RNA 6000 Nano Kit (reorder number 5067–1511) was employed to detected the RNA integrity. Subsequently, Clariom™ S Array mouse (*TermoFisher, Madrid, Spain*) was used to conduct the Microarray assay according to standard protocols. All Microarray assay was performed in the Unidad de Genómica, CAI Genómica y Proteómica, Facultad CC Biológicas. UCM. Transcriptome Analysis Console (*TermoFisher, Madrid, Spain*) was employed to commit the analysis. Probes were assigned to unique gene identifiers according to the official gene symbol. Robust Multi-array Average (RMA) method were implied to normalize the arrays. Subsequently statistical analysis were performed with eBayes (limma).

1.12. Western Blot

Protein contents were measured using Protein Assay Dye Reagent Concentrate (Bio-Rad, California, USA) according to manufacturer`s protocol. Samples were separated in 10% SDS-PAGE polyacrylamide gel (BioRad, California, USA) and blotted into a polyvinylidene fluoride membrane (BioRad, California, USA) by transference blotting. Membranes were then blocked and incubated with primary and then secondary antibodies (Table S8). Enhanced Chemiluminescence (ECL; Merck, Munich, Germany) method was used to detect protein bands.

1.13. Magnetic Resonance Imaging (MRI)

The whole study has been carried out on an MRI Biospin 7T equipment (Bruker, Germany) using a volume antenna. Two sequences, are acquired with and without fat suppression. The parameters of these sequences are: TE/TR = 6.5/1500 ms. 2 averages. Rare factor 4. Coronal. Select the number of slices and adjust to the size of the animal. Fov = 80 × 40 mm. Matrix 256 × 256 pixels. Slice thickness = 0.5 mm. Slice gap = 0.25 mm. The estimated value of fat in the body, was obtained by using ImageJ (ImageJ 1.53e. Wayne Rasband and contributors National Institute of Health, USA) software. The fat- free image is subtracted from the fat-non suppressed image to obtain a fat-only image. After a noise filtering process, the volume of segmented fat is measured and, using a density of 0.9 g/mL, and an estimation of the fat mass is obtained.

After homogenizing the magnetic field, a proton spectrum of the entire body of the animal is acquired using a PRESS_1H sequence with the following parameters: TE/TR = 16.5/2500 ms and 64 averages.

The analysis was carried out with the MNova software (Mestrelab, Spain). The area under the curve (AUC) of the water and fat peaks was measure, as well as the intensity of these peaks, and the relationship between peaks of these values is calculated.

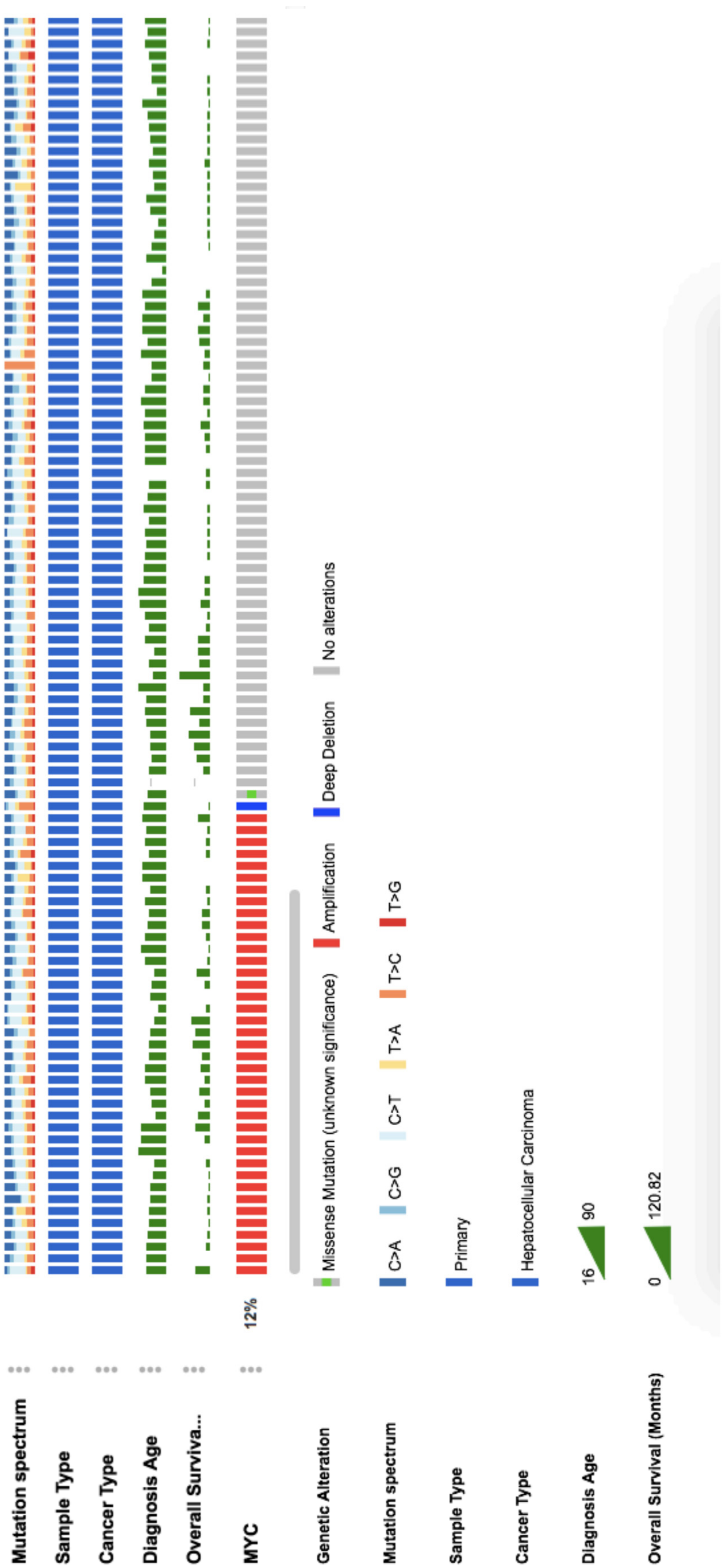
Mutation spectrum
Sample Type
Cancer Type
Diagnosis Age
Overall Surviva...
MYC
12%
Genetic Alteration
Missense Mutation (unknown significance)
Amplification
Deep Deletion
No alterations
Mutation spectrum
C>A
C>G
C>T
T>A
T>C
T>G
Sample Type
Primary
Cancer Type
Hepatocellular Carcinoma
Diagnosis Age
16
90
Overall Survival (Months)
0
120.82

Figure S1. Bioinformatic analysis of 353 human HCC samples based on data generated by the Cancer Genome Atlas (TCGA) Research Network.

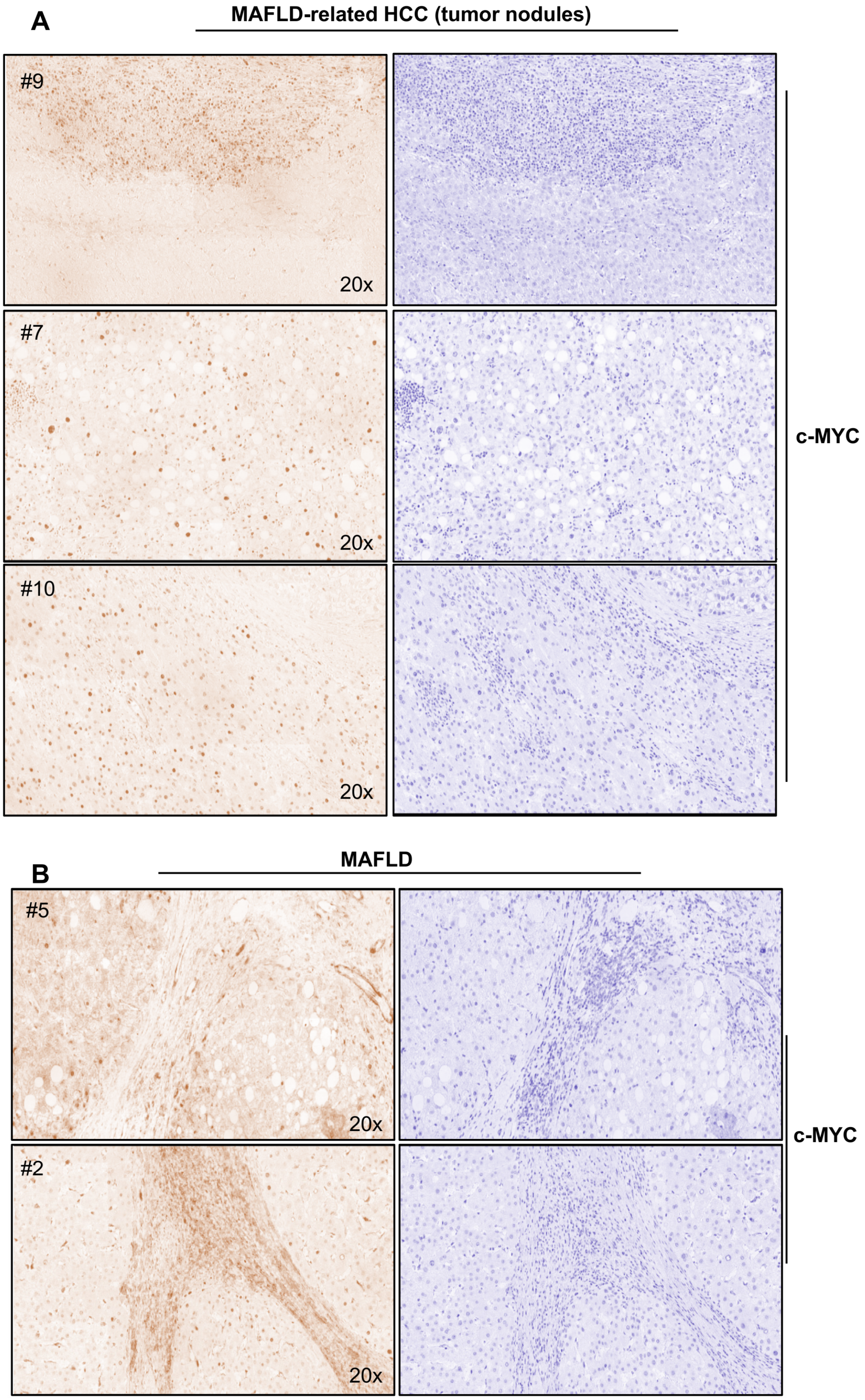

Figure S2. Representative IHC-stained sections demonstrating nuclear expression of c-MYC. **(A)** c-MYC nuclear expression inside tumor nodules in patients with MAFLD-related HCC. Left: the DAB-stained images. Right: Hematoxylin stained images. (**B**) c-MYC nuclear expression in MAFLD

patients with advanced liver fibrosis. Left: the DAB-stained images. Right: Hematoxylin stained images.

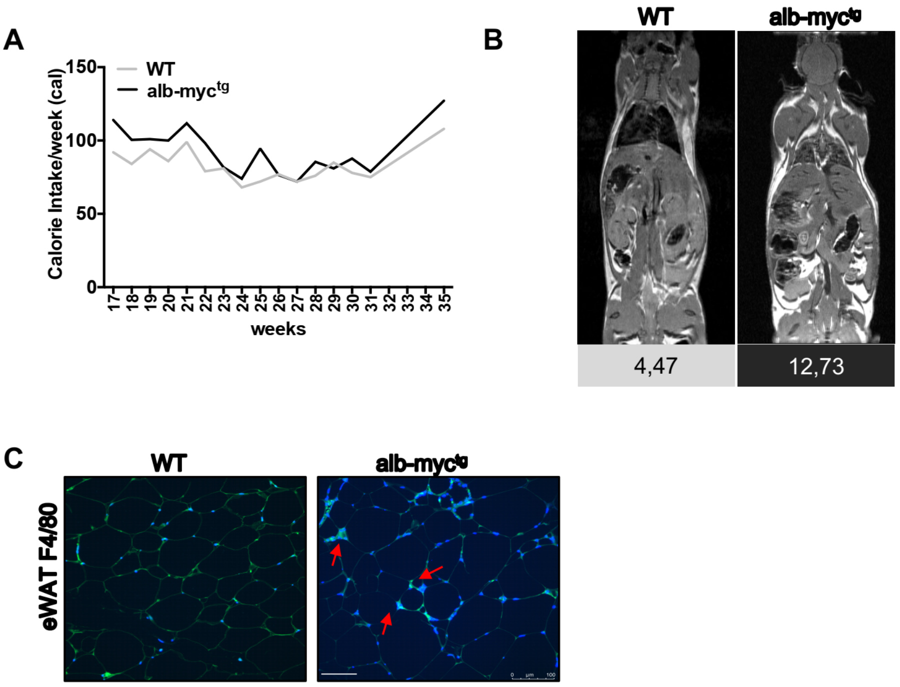

Figure S3. MS in alb-myc^{tg} at baseline. WT (n = 9) and alb-myc^{tg} (n = 8) mice at the age of 36 weeks. **(A)** Food caloric intake was stable in both groups through the duration of study. **(B)** Representative MRI scan images of WT and alb-myc^{tg} mice showing body fat distribution in 26 weeks old animals. The quantitative data represent fat/body ratio (%) means. **(C)** Representative F4/80 IF staining of WAT. Positive cells are stained in green and marked with red arrows. Nuclei are stained in blue using DAPI as counterstain. Scale bar is 100 µm (n = 4).

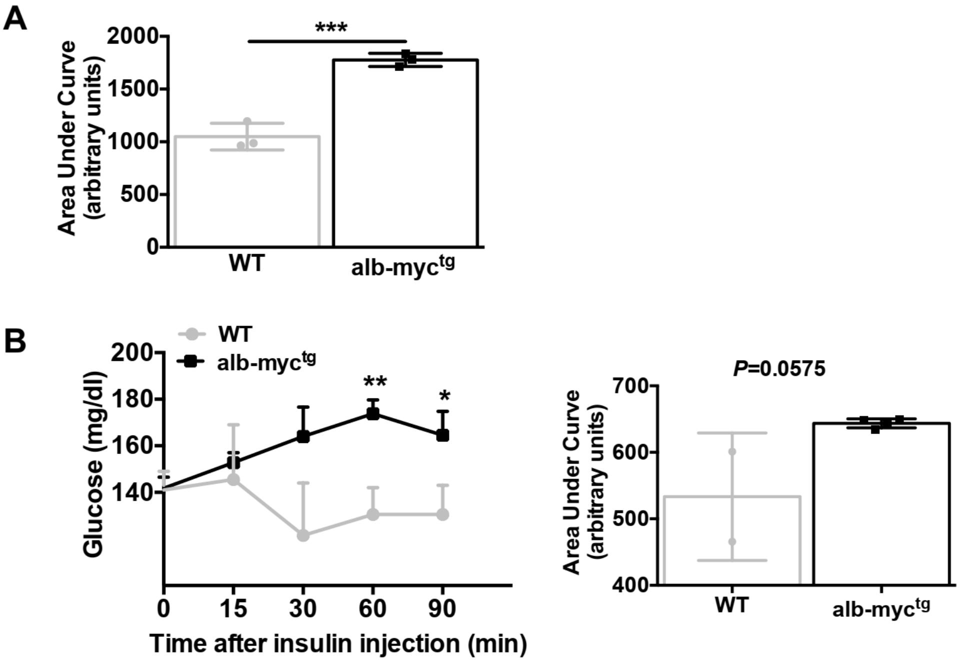

Figure S4. Glucose intolerance. **(A)** The histogram represents the incremental area under the respective glucose tolerance curve (n = 3). **(B)** Insulin tolerance test (ITT), and the histogram represents the incremental area under the curve (n = 3). Data are expressed as the mean ± SD, *=p<0.05; **=p<0.01, alb-myc^{tg} mice vs. WT controls.

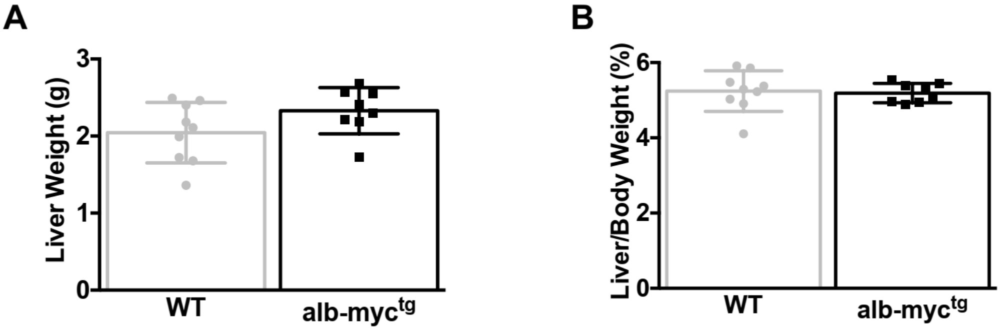

Figure S5. Hepatosomatic ratio at 36 weeks. WT (n = 9) and alb-myc^{tg} (n = 8) mice at the age of 36 weeks. **(A)** Liver weight. **(B)** Liver to body weight ratio.

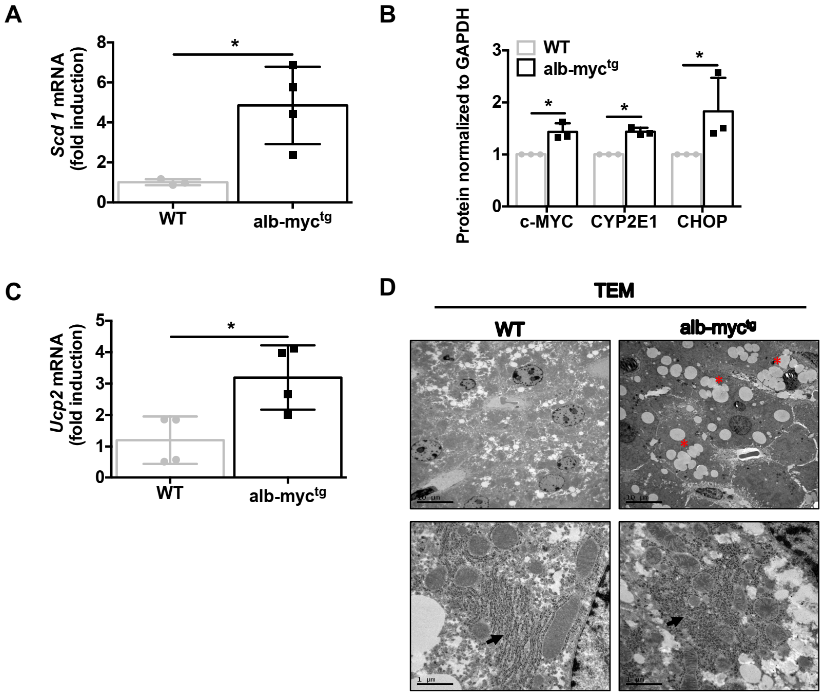

Figure S6. Hepatic phenotype in WT and alb-myc^{tg} mice at 36 weeks. **(A)** qPCR analysis of hepatic mRNA expression expression of *Scd1.* **(B)** Histograms showing densitometric analysis of western blots for c-MYC, CYP2E1 and CHOP. **(C)** qPCR analysis of hepatic mRNA expression expression of *Ucp2* (n = 4). **(D)** Representative TEM images of hepatic endoplasmic reticulum. Representative Transmission Electro-Microscopy (TEM) pictures of WT and alb-myc^{tg} mice. Asterisks indicate fat droplets in hepatocytes. Arrows mark endoplasmic reticulum (ER). Scale= 10μm and 1 μm.

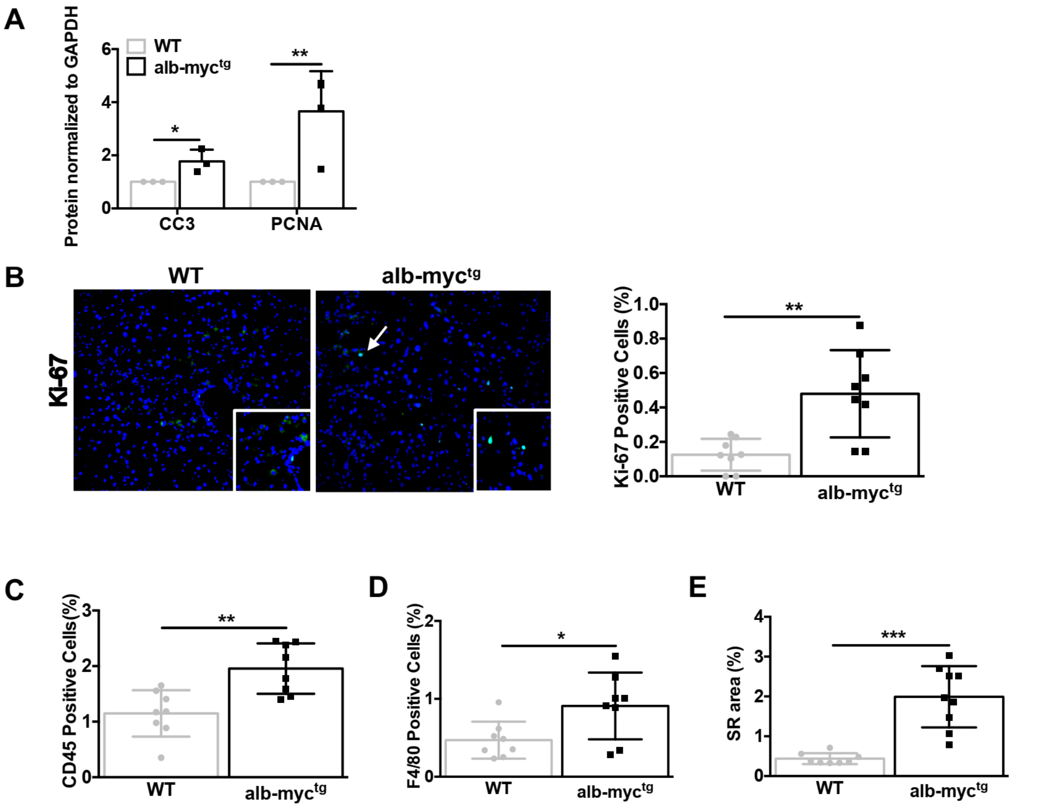

Figure S7. Spontaneous proliferation and mild inflammation in alb-myc^{tg} mice at 36 weeks. **(A)** Histograms showing densitometric analysis of western blots for CC3 and PCNA. **(B)** Ki-67 immunofluorescence of crytosections showing increased cell proliferation (green, as indicated by arrow) of hepatocytes in alb-myc^{tg} mice liver and the quantification of % Ki-67 positive cells (n = 8). **(C-E)** Quantification of % CD45, F4/80- positive cells and SR area respectively using ImageJ software (n = 8). Data are expressed as the mean ± SD, *=p<0.05; **=p<0.01, ***=p<0.001, alb-myc^{tg} mice vs. WT controls.

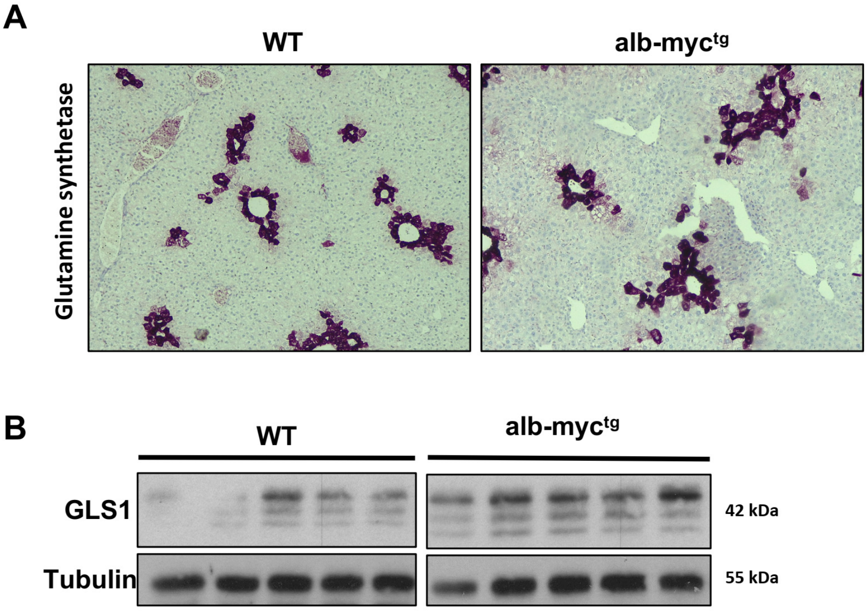

Figure S8. Increased glutamine catabolism in alb-myc^{tg} mice at 36 weeks. (n = 5). **(A)** IHC staining of Glutamine synthetase (GS). **(B)** Protein expression of Glutaminase 1 (GLS1) in the livers of alb-myc^{tg} and WT mice.

A

Top molecules	
WT *vs.* alb-myc[tg]	
**Upregulated**	**Downregulated**
**ER stress**<br>*ORM3, CXCL1*	**Metabolism**<br>*SLCO1A1, HSD3B4, HSD3B5, ELOVL3*
**Lipid/fatty acid metabolism**<br>*Fabp4, aP2, SLUT1E1*	
**Glucose homeostasis**<br>*PFKFB1, PFKFB3*	
**Cytochrome P450**<br>*CYP2B10, Por*	
**DNA damage**<br>*DDIT4, CDKN1A, GADD45G*	
**Oncogene**<br>*MYC*	

B

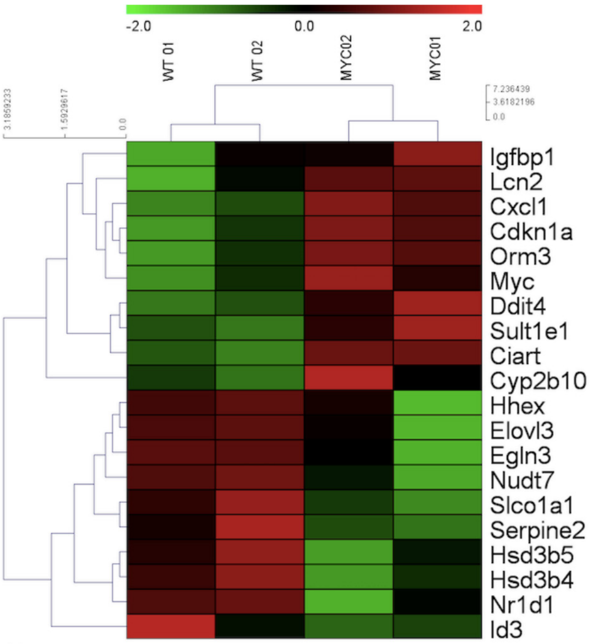

C

Top canonical pathways
WT *vs.* alb-myc[tg]
**Cytochrome P450 panel**
**Lipid/fatty acid metabolism**
**ER stress**
**Glucose homeostasis**
**Hepatotoxicity**

D

Hepatotoxicity

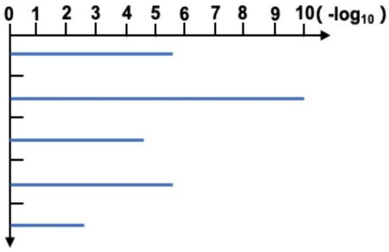

Name	molecules
Liver steatosis	25
Liver proliferation	15
Liver damage	15
Liver inflammation/hepatitis	20
Liver regeneration	7

Figure S9. Activation of signaling pathways relevant to human MAFLD in the liver of mice. **(A)** Dysregulated genes involved in different pathways. **(B)** Heat map demonstrating deregulated genes. Red and green colors indicate high and low gene expression, respectively. **(C)** Top canonical pathways. **(D)** Molecules involved in hepatotoxicity. All data are based on statistical significance.

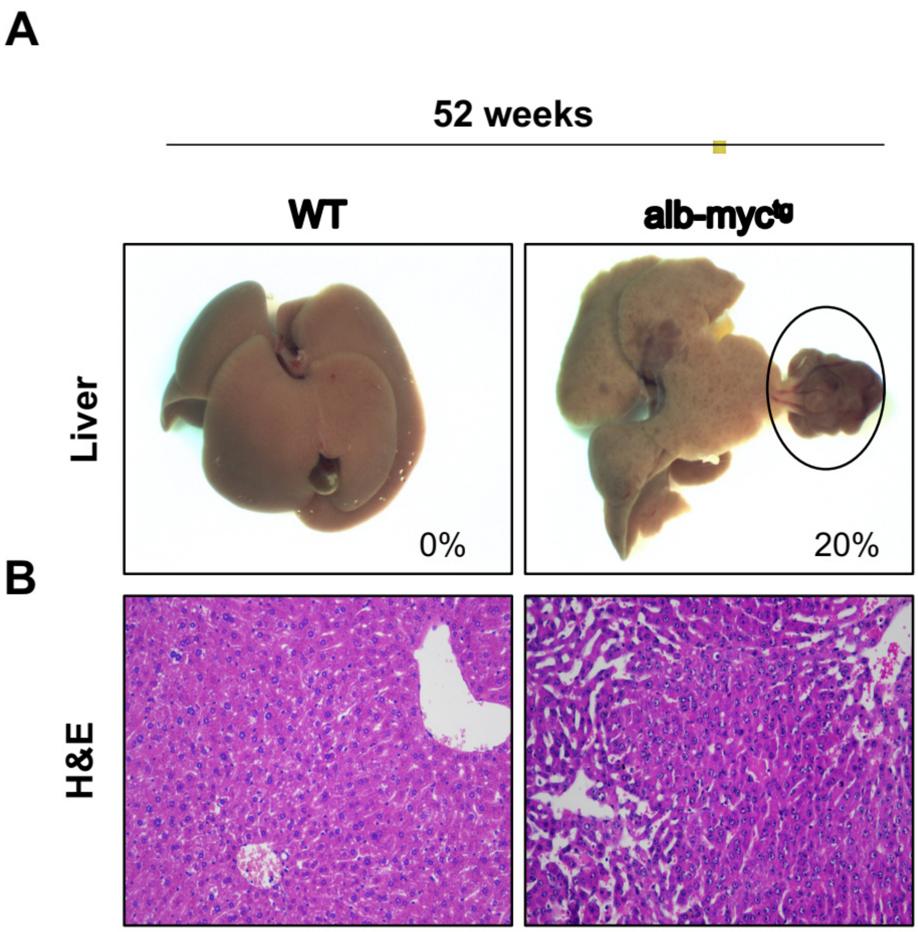

Figure S10. Spontaneous HCC development in alb-myc^{tg} mice. WT (n = 3) and alb-myc^{tg} (n = 5) mice at the age of 52 weeks. **(A)** Macroscopic images of the liver. Numbers represent tumor incidence (%) means **(B)** Representative liver sections stained with H&E.

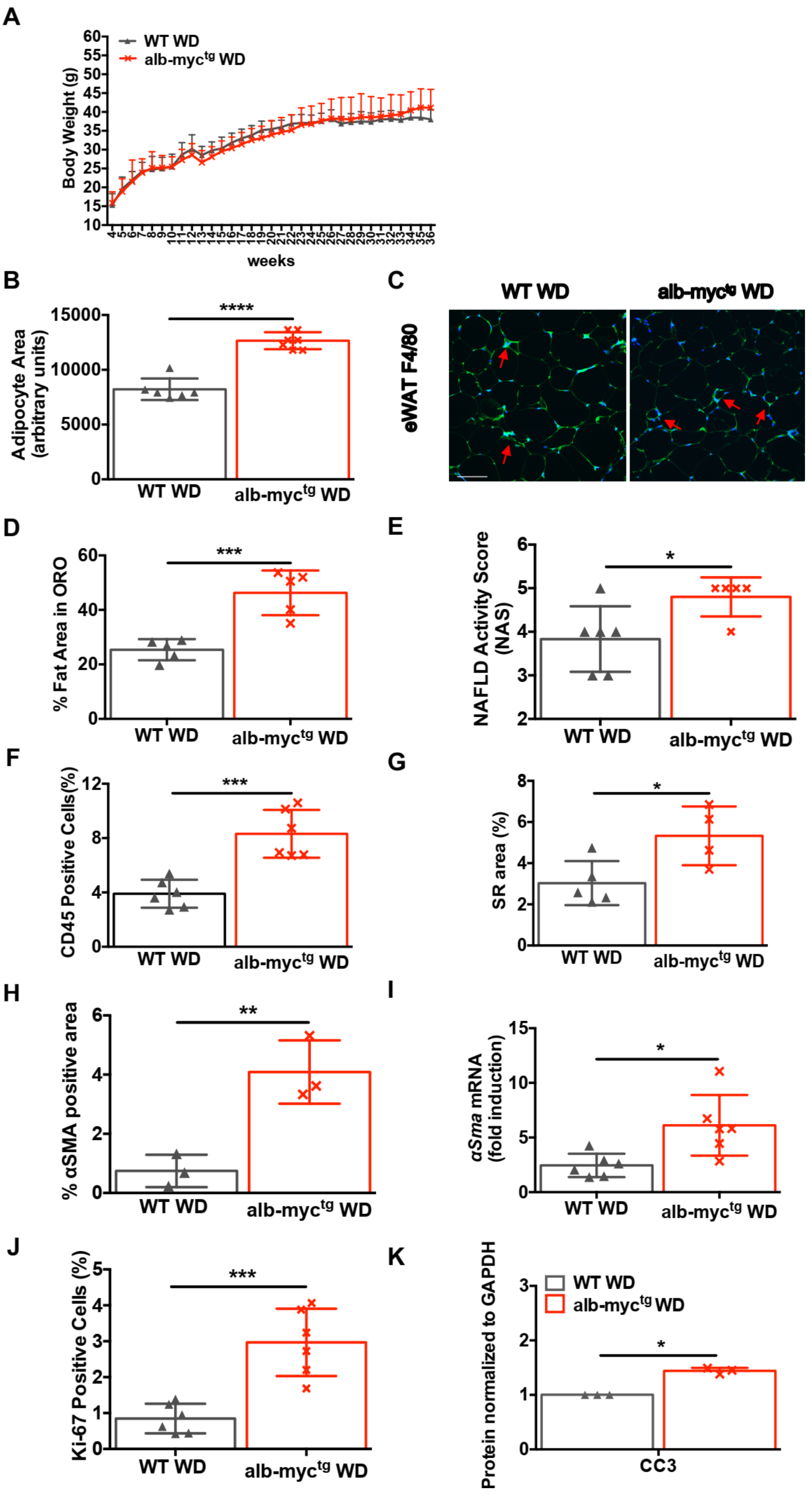
A
WT WD
alb-myc^{tg} WD
Body Weight (g)
weeks
B
****
Adipocyte Area
(arbitrary units)
WT WD
alb-myc^{tg} WD
C
WT WD
alb-myc^{tg} WD
eWAT F4/80
D
***
% Fat Area in ORO
WT WD
alb-myc^{tg} WD
E
*
NAFLD Activity Score
(NAS)
WT WD
alb-myc^{tg} WD
F
***
CD45 Positive Cells(%)
WT WD
alb-myc^{tg} WD
G
*
SR area (%)
WT WD
alb-myc^{tg} WD
H
**
% αSMA positive area
WT WD
alb-myc^{tg} WD
I
*
αSma mRNA
(fold induction)
WT WD
alb-myc^{tg} WD
J
***
Ki-67 Positive Cells (%)
WT WD
alb-myc^{tg} WD
K
Protein normalized to GAPDH
WT WD
alb-myc^{tg} WD
*
CC3

Figure S11. Phenotypical changes in WT and transgenic mice fed with WD for 24 weeks. **(A)** Growth curve demonstrating body weight gain, assessed at 7 days interval at each group **(B)** Relevant adipocyte area of eWAT (*n* = 6). **(C)** Representative F4/80 IF staining of WAT. Positive cells are stained in green and marked with red arrows. Nuclei are stained in blue using DAPI as counterstain. Scale bar is 100 µm (*n* = 4). **(D)** Quantification of Oil Red O positive area (*n* = 5). **(E)** Histological NAFLD activity score (*n* = 5). **(F)** Quantification of % CD45 positive cells (*n* = 6). **(G)** Quantification of % SR positive area (*n* = 4). **(H)** Quantification of % αSMA positive area (*n* = 3). **(I)** qPCR analysis of hepatic tissue of fibrosis gene *αSma* (*n* = 6). **(J)** Quantification of % KI-67 positive cells (*n* = 6). **(K)** Histograms showing densitometric analysis of western blot for CC3. Data are expressed as the mean ± SD, *=p<0.05; **=p<0.01, ***=p<0.001, alb-myc^{tg} mice vs. WT animals fed WD.

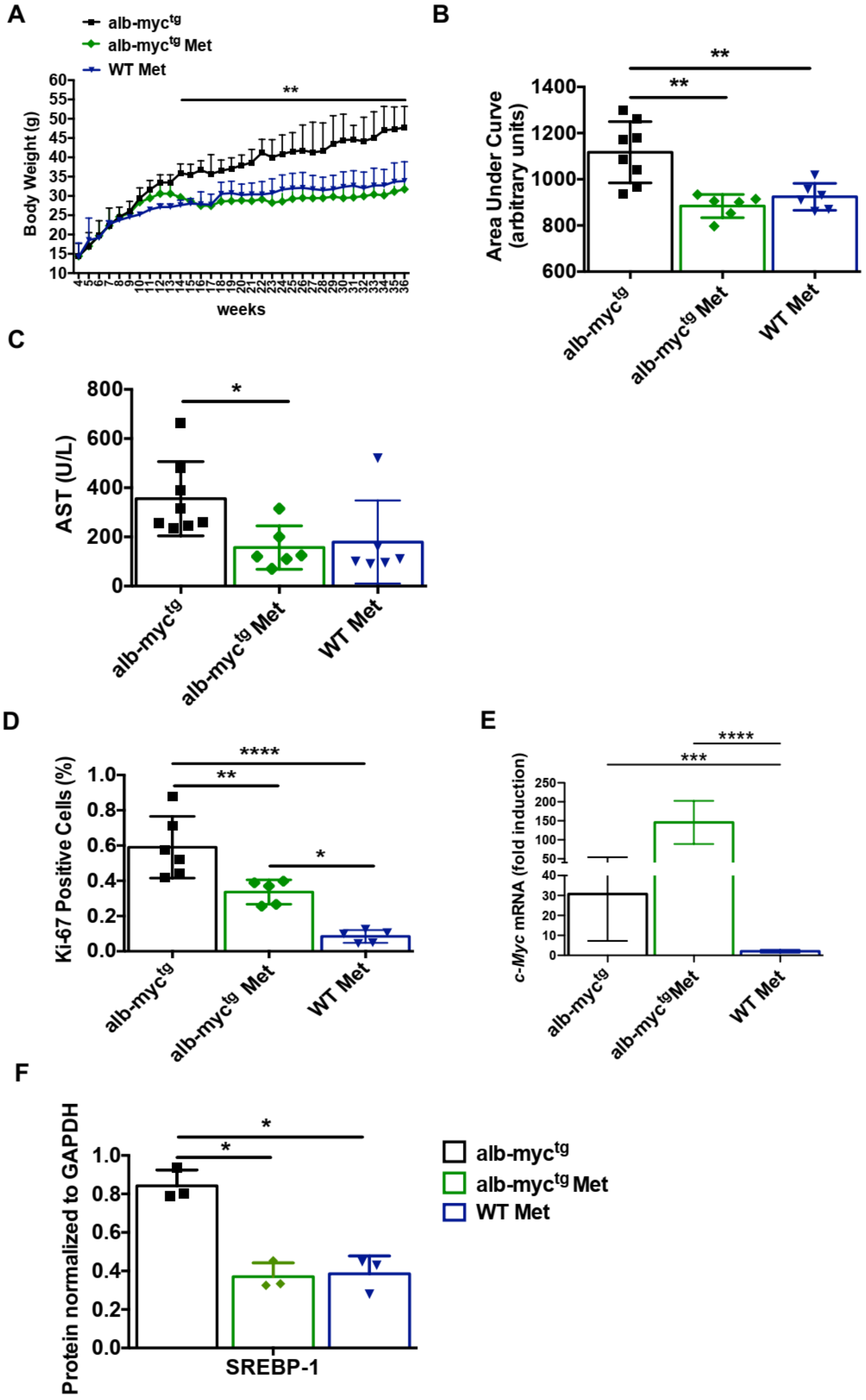

Figure S12. MS and phenotypical changes in animals fed with metformin enriched diet. **(A)** Growth curve demonstrating body weight gain of mice, assessed at 7 days interval at each group, and **(B)** Relevant histogram represents the incremental area under the respective body weight curve. **(C)** Level of AST in serum (n = 6–8). **(D)** Quantification of % Ki-67 positive cells (n = 6). **(E)** qPCR analysis of hepatic mRNA expression expression of *c-Myc.* **(F)** Histograms showing densitometric analysis of western blots for SREBP1. Data are expressed as the mean ± SD, *=p<0.05, **=p<0.01, ***=p<0.001, alb-myc^{tg} mice fed metformin enriched chow diet vs. alb-myc^{tg} mice fed chow diet; *=p<0.05, **=p<0.01, ***=p<0.001, ****=p<0.0001, WT mice fed metformin enriched chow diet vs. alb-myc^{tg} mice fed chow diet.

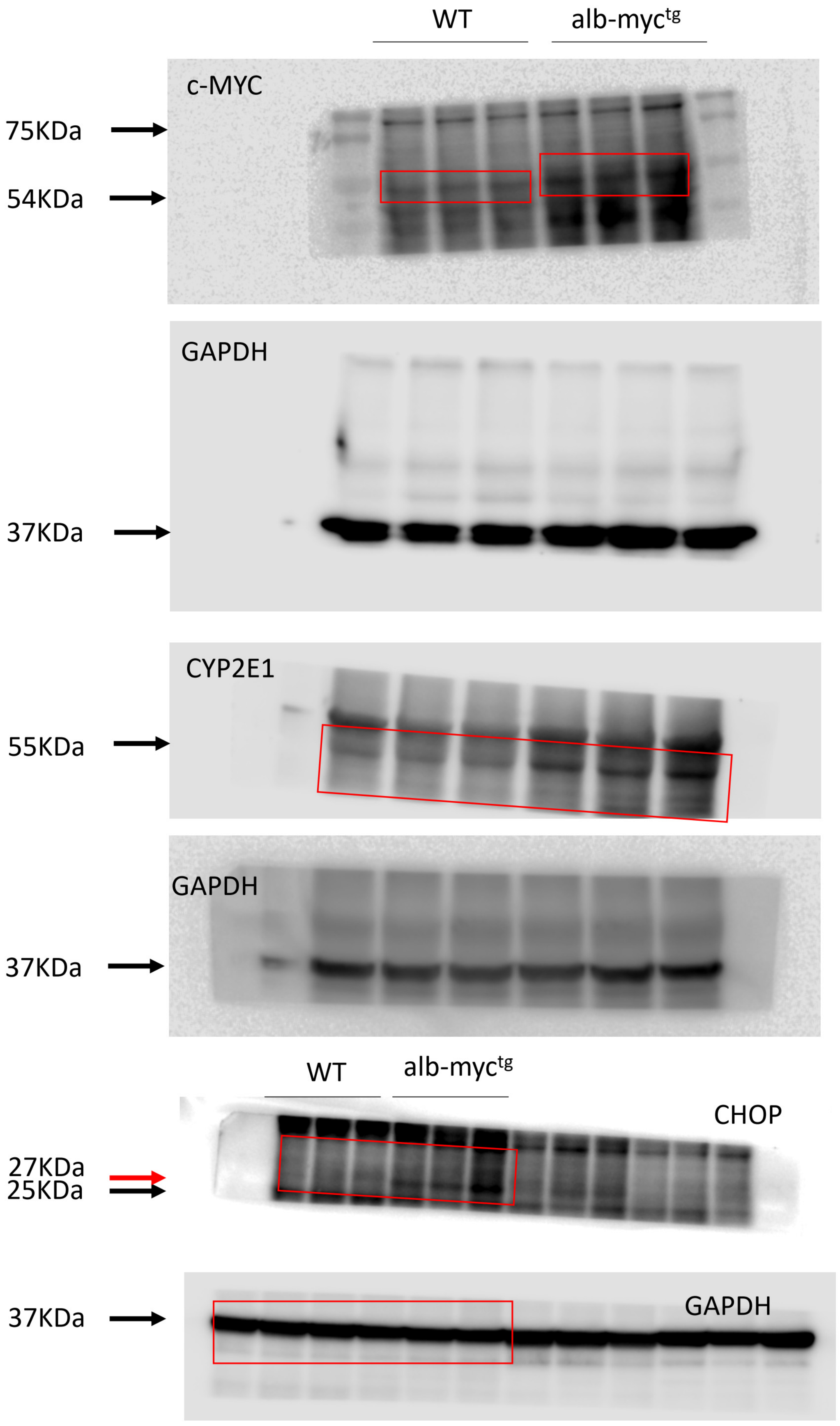

Figure S13. Uncropped blots for Figure 4 C.

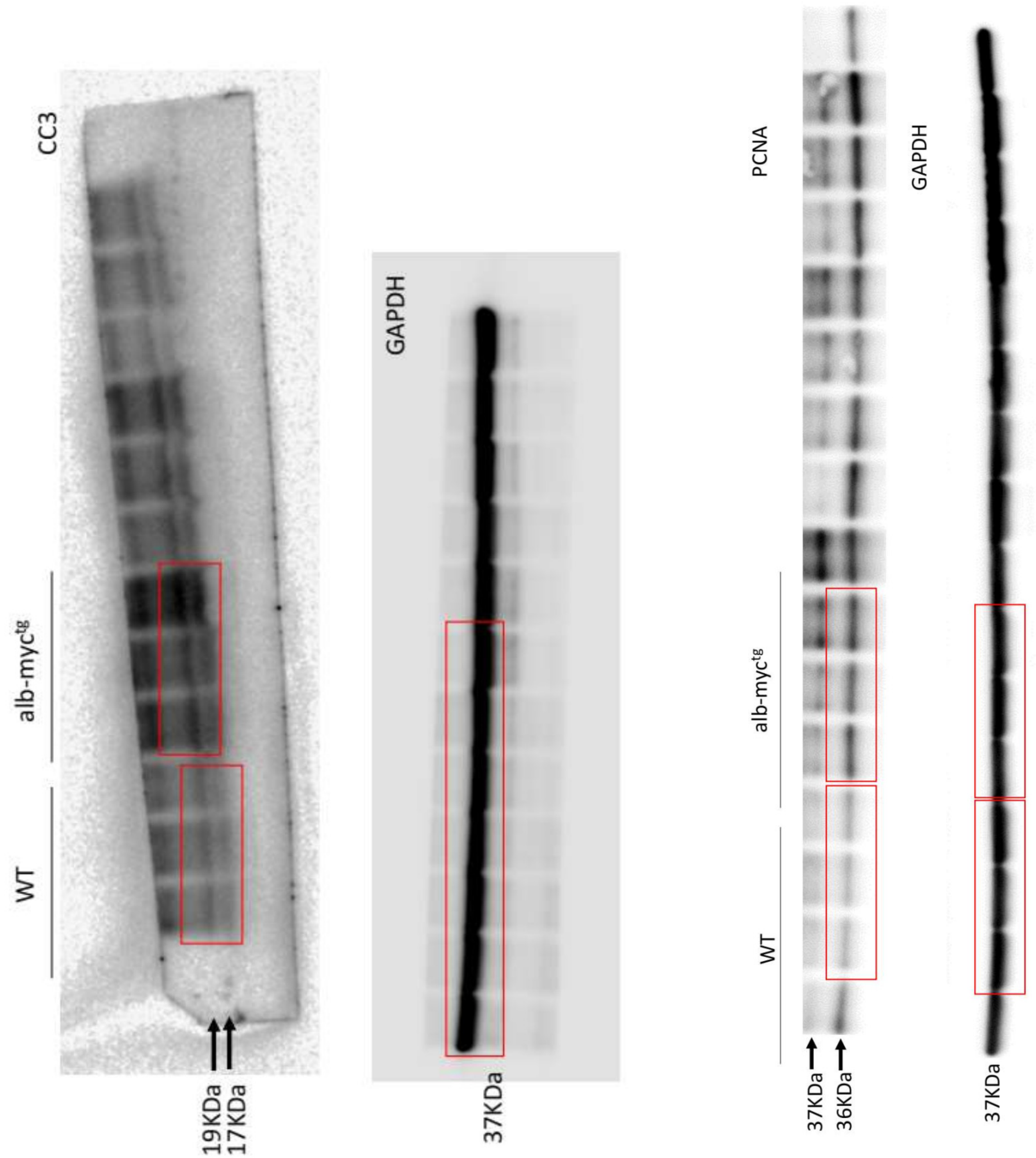

Figure S14. Uncropped blots for Figure 4 E.

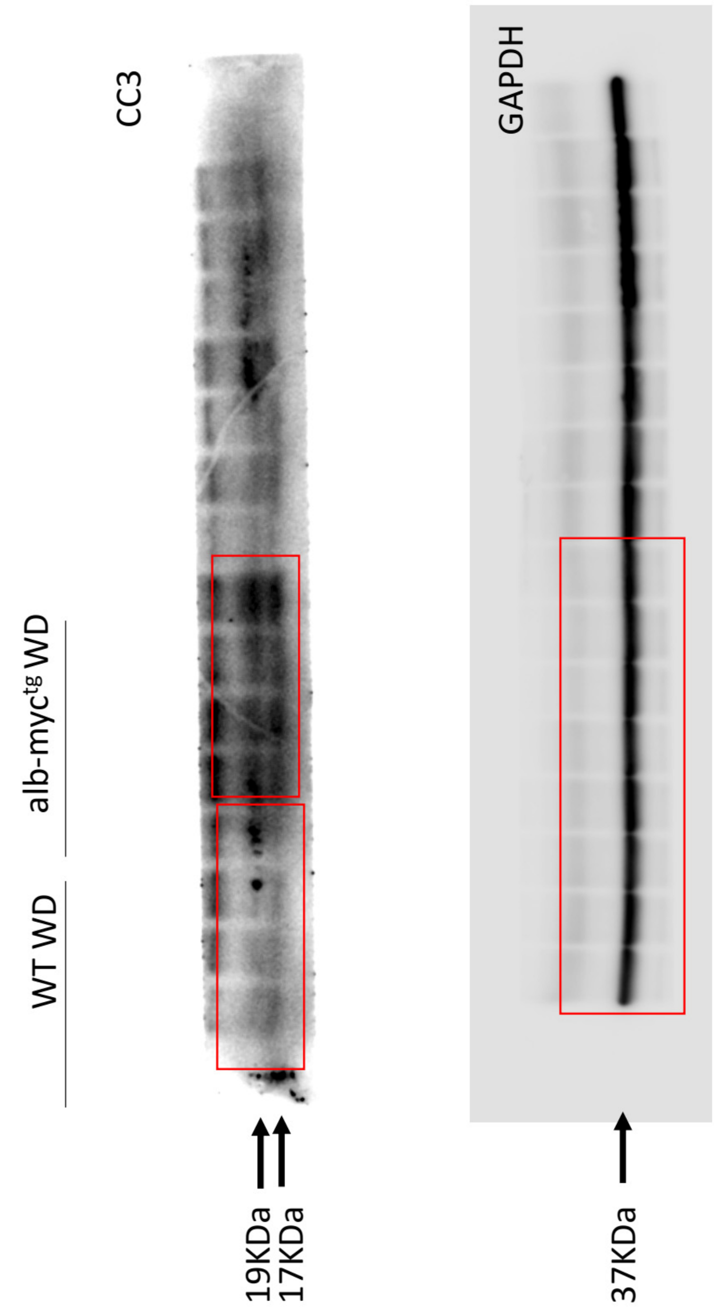

Figure S15. Uncropped blots for Figure 5 I.

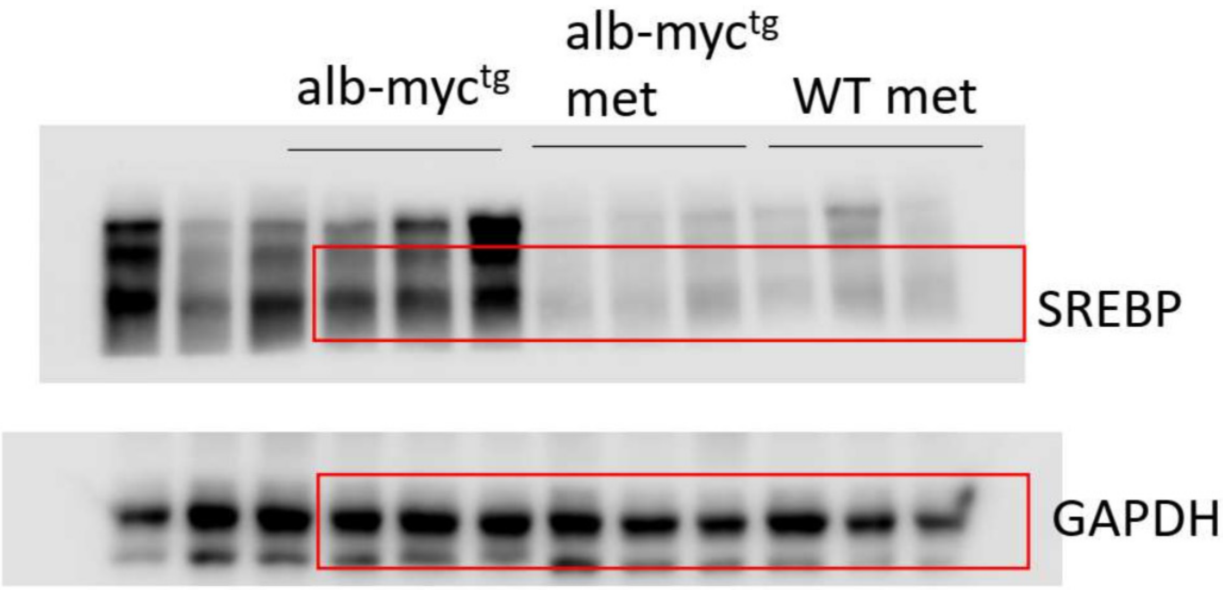

Figure S16. Uncropped blots for Figure 7I.

Table S1. Etiology of human liver samples used for c-MYC gene expression analysis.

Sample	Age (years)	Sex	Grade of fibrosis	Etiology
#1	42	1	F4	MAFLD
#2	57	0	F4	MAFLD
#3	59	0	F4	MAFLD
#4	72	0	F3	MAFLD
#5	64	0	F3	MAFLD
#6	50	0	F3-F4	MAFLD
#7	69	0	F0	MAFLD+HCC
#8	72	0	F0	MAFLD+HCC
#9	66	1	F4	MAFLD+HCC
#10	52	0	F0	MAFLD+HCC
#11	74	1	F4	MAFLD+HCC

Abbreviation: Sex: 0 = male; 1= female.

Table S2. Clinical features of human liver samples.

		**MAFLD** *N* = 6	**MAFLD HCC** *N* = 5	**STATISTICS**
	AGE (mean + SD)	57 ± 10	67 ± 9	ns
	SEX (Female/Male)	5/1	3/2	ns
	FIBROSIS STAGE 0/1/2/3/4	0/0/2/4	3/0/2	ns
	Transplanted no/yes	4/2	3/2	ns
TUMOUR FEATURES	Edmonson I/II/III/IV	-	0/4/1/0	-
	Treatment No/yes * previous resection	-	4/1*	-
	BCLC A/B/C/D	-	5/0/0/0	-

	METASTASIS no/yes	-	5/0	-
CLINICAL PARAMETERS	DIABETES no/yes *1 prediabetes	2/4	3*/2	ns
	HTA no/yes	4/2	0/5	0.022
	DISLIPEMIA no/yes	2/4	3/2	ns
	OBESITY (BMI ≥ 30) no/yes	2/4	2/3	ns
BIOCHEMICAL PARAMETERS	AST U/L (mean +SD) * 1 missing value	39 ± 22	23±22*	ns
	ALT U/L (mean +SD) *High heterogeneity 1 Patient from HCC has 273 Median values for each category (45/52)	43 ± 18	89 ± 106*	ns
	GGT U/L (mean +SD) * 1 missing value	290 ± 258	45 ± 49*	ns

Statistical significance was determined by Pearson Chi Square 2 sided or *t*-test independent samples 2 tails (age, AST, ALT, GGT).

Table S3. Kcal% of chow diet (CD) and western diet (WD).

Nutrients	CD	WD
Protein	18.9%	20%
Fat	5.3%	40%
Others	75.8%	40%

Table S4. Diets used for the development of MAFLD.

DIETS	CATEGORY NO.	COMPANY
Chow diet	LASQC diet® Rod18-H	Altromin, Lage，Germany
Western diet	D09100301	Research Diets, New Brunswick, NJ
Chow diet enriched 0.1% metformin	C19051603	Research Diets, New Brunswick, NJ

Table S5. Antibodies used for IF staining.

Antibody	Reference/Company	Dilution
Rat Anti-mouse CD45	550539 BD Pharmingen (San Diego, CA)	1:100
Rat Anti-mouse F4/80	MGCA497GA BIO-RAD (Hercules, CA)	1:100–1:150
Rabbit Anti-mouse Ki67	Ab16667 Abcam (Burlingame, CA)	1:200
Alexa Fluor® 488 goat anti-rat IgG (H+L)	A11006 Invitrogen (Paisley, UK)	1:500
Alexa Fluor® 488 donkey anti-rabbit IgG (H+L)	A21206 Invitrogen (Paisley, UK)	1:400

Table S6. Antibodies used for IHC and IF stainings.

Antibody	Reference/Company	Dilution
Rabbit Anti Mouse Ki-67	Ab16667 Abcam (Burlingame, CA)	1:100
Rabbit Anti Mouse αSMA	Ab32575 Abcam (Burlingame, CA)	1:500
Rabbit Anti-c-MYC	Ab32072 Abcam (Burlingame, CA)	1:100
Glutamine synthetase	Sc74430 Santa Cruz Biotechnology (Dallas, TX)	1:200
HRP Anti-Rabbit IgG (Peroxidase) Polymer Detection Kit made in horse	MP-7401 Vectorlab (Burlingame, CA)	-

Table S7. Primers for qPCR.

	**Primers for mouse**	
**Gene**	**Forward**	**Reverse**
Acadm	GCCAACTGGTATTTCTTGTTGG	CTGGCCCATGTTTAGTTCCT
αSma	GACAGAGGCACCACTGAACC	TCCAGAGTCCAGCACAATACCAGT
Cpt 1	GCATGGTAGATGTTTCGACAG	GCCATGACATACTCCCACAG
Gapdh	TGTTGAAGTCACAGGAGACAACCT	AACCTGCCAAGTATGATGACATCA
Scd1	GTTCCAGAATGACGTGTACGA	GGCTTGTAGTACCTCCTCTG
Ucp 2	TGGAAAGGGACTTCTCCCA	GGGAGGTCATCTGTCATGAG
c-Myc	AGTGCTGCATGAGGAGACAC	GGTTTGCCTCTTCTCCACAG

Table S8. List of antibodies used for WB.

Antibody	Reference/Company	Dilution
Anti-Rabbit HRP	7074 Cell Signaling（Denver, USA）	1:3000
CHOP	2895s Cell Signaling（Denver, USA）	1:1000
CLEAVED CASPASE 3	9661L Cell Signaling（Denver, USA）	1:800
c-MYC	Ab32072 Abcam (Burlingame, CA)	1:100
CYP2E1	Ab28146 Abcam (Burlingame, CA)	1:4000
GAPDH	MCA4739 BIO-RAD (Hercules, CA)	1:5000
GLS1	AGIOS Pharmaceuticals (Cambridge, MA)	1:1000
Goat Anti-mouse HRP	STAR207P BIO-RAD (Hercules, CA)	1:5000
PCNA	13-3900 Invitrogen (Paisley, UK)	1:1000
TUBULIN	T8328 Sigma (St. Louis, MO)	1:10000
SREBP1	Sc17755 Santa Cruz Biotechnology (Dallas, TX)	1:200

References

1. Clapper, J.R.; Hendricks, M.D.; Gu, G.; Wittmer, C.; Dolman, C.S.; Herich, J.; Athanacio, J.; Villescaz, C.; Ghosh, S.S.; Heilig, J.S. Diet-induced mouse model of fatty liver disease and nonalcoholic steatohepatitis reflecting clinical disease progression and methods of assessment. *American Journal of Physiology-Gastrointestinal and Liver Physiology* **2013**, *305*, G483–G495.
2. Benedé-Ubieto, R.; Estévez-Vázquez, O.; Ramadori, P.; Cubero, F.J.; Nevzorova, Y.A. Guidelines and Considerations for Metabolic Tolerance Tests in Mice. *Diabetes, Metabolic Syndrome and Obesity: Targets and Therapy* **2020**, *13*, 439.

3. Mehlem, A.; Hagberg, C.E.; Muhl, L.; Eriksson, U.; Falkevall, A. Imaging of neutral lipids by oil red O for analyzing the metabolic status in health and disease. *Nature protocols* **2013**, *8*, 1149–1154.
4. Nevzorova, Y.A.; Hu, W.; Cubero, F.J.; Haas, U.; Freimuth, J.; Tacke, F.; Trautwein, C.; Liedtke, C. Overexpression of c-myc in hepatocytes promotes activation of hepatic stellate cells and facilitates the onset of liver fibrosis. *Biochim Biophys Acta* **2013**, *1832*, 1765–1775, doi:10.1016/j.bbadis.2013.06.001.
5. Benede-Ubieto, R.; Estevez-Vazquez, O.; Guo, F.; Chen, C.; Singh, Y.; Nakaya, H.I.; Gomez Del Moral, M.; Lamas-Paz, A.; Moran, L.; Lopez-Alcantara, N.; et al. An Experimental DUAL Model of Advanced Liver Damage. *Hepatol Commun* **2021**, *5*, 1051–1068, doi:10.1002/hep4.1698.
6. Nevzorova, Y.A.; Bangen, J.M.; Hu, W.; Haas, U.; Weiskirchen, R.; Gassler, N.; Huss, S.; Tacke, F.; Sicinski, P.; Trautwein, C. Cyclin E1 controls proliferation of hepatic stellate cells and is essential for liver fibrogenesis in mice. *Hepatology* **2012**, *56*, 1140–1149.
7. Livak, K.J.; Schmittgen, T.D. Analysis of relative gene expression data using real-time quantitative PCR and the 2(-Delta Delta C(T)) Method. *Methods* **2001**, *25*, 402–408, doi:10.1006/meth.2001.1262.
